# Development and Analysis of High-Modulus Asphalt Concrete Predictive Model

**DOI:** 10.3390/ma16134509

**Published:** 2023-06-21

**Authors:** Mikołaj Bartkowiak, Mieczysław Słowik

**Affiliations:** Faculty of Civil and Transport Engineering, Poznan University of Technology, Piotrowo 3, 60-965 Poznań, Poland

**Keywords:** high-modulus asphalt concrete, bitumen, stiffness modulus |E*|, shear modulus |G*|, four-point bending beam test, predictive model, optimization

## Abstract

The main purpose of this paper is to present the development of a new predictive model intended for the calculation of stiffness modulus |E*| determined by a four-point bending beam test (4PBB or 4PB-PR). The model developed, called model A, was based on the Witczak model, which was developed for the dynamic-modulus (DM) method. Most of the asphalt mixtures used to develop the model were high-modulus asphalt concrete (HMAC). The most commonly used methods for determining the stiffness modulus |E*| of asphalt mixtures were also discussed. The paper presents the results of the study for 10 asphalt mixtures but 8 of them were used to develop the predictive model. In addition, the results of complex shear modulus G* tests on neat and modified bituminous binders carried out in a dynamic shear rheometer (DSR), necessary for the development of a predictive model, are presented. The tests carried out in the dynamic shear rheometer had significant measurement uncertainties. The results of the volumetric parameters of the asphalt mixtures are also reported. The developed model A has maximum absolute errors e = 1930 MPa (*p* = 95%) and maximum relative errors re = 50% (*p* = 95%). The distribution of the absolute errors of the model, after discarding outliers, has a normal distribution as in the development of other models of this type, which was confirmed by appropriate statistical tests. On the basis of the tests and calculations carried out, it was concluded that, in order to increase the precision of the predictive models, it is advisable to reduce the measurement uncertainty of the bitumen complex shear modulus G*. For the developed model A, the limiting values of the stiffness modulus |E*| are also shown, within which the determined stiffness modulus should fall.

## 1. Introduction

The stiffness modulus |E*| of an asphalt mixture is an important parameter characterizing its properties. Different values of stiffness modulus |E*| appear at particular stages of asphalt pavement design and realization. The stiffness modulus |E*| is used in the designing process of pavements through mechanistic methods. It is also determined in the case of asphalt mixture composition designing as well as the factory production-control process [1].

The stiffness modulus |E*| is a physical property describing the relationship between stress and strain in the case of linear viscoelastic material as it is loaded and unloaded. When the stress and stiffness modulus are known, the strain and displacement can be calculated. The stiffness modulus |E*| extensively describes the asphalt mix, due to its dependence on many factors. These factors are the composition of an asphalt mixture, the content of air voids, shape, lay down, and composition of aggregate grains in an asphalt mixture. The values of the stiffness modulus |E*| change substantially along with the temperature and loading frequency [2,3] which makes the cause of differentiation between the stiffness modulus |E*| and modulus of elasticity. The value of stiffness modulus |E*| depends on the method of determining, testing equipment, conditions during the tests, and also on the shape and dimensions of the tested samples. The procedure of making samples or sampling and their age [4,5] are also important factors.

In Europe, stiffness modulus is determined mainly with the use of tests described in the standard EN 12697-26 [6]. In the United States and other countries where the *Mechanistic—Empirical Pavement Design Guide* (MEPDG) [7] is used for designing pavements, the stiffness modulus is defined according to the standard of American Association of State Highway and Transportation Officials: AASHTO T 342-11 Determining Dynamic Modulus of Hot Mix Asphalt (HMA) [8]. The stiffness modulus values obtained by laboratory methods should be correlated with tests performed on actual pavements [9,10].

In a nutshell, the stiffness modulus |E*| may be considered the most important property of an asphalt mixture. For this reason, a large number of predictive models for the calculation of the stiffness modulus have been developed over the years, using different data characterizing the asphalt mixture. The different predictive models are discussed in [11]. One of the first models was proposed by Van der Poel in 1954 [12]. The most commonly used models are the Witczak model, the Hirsch model, and models using artificial neural networks (ANN models). The models currently in use make it possible to calculate the value of the stiffness modulus for a wide range of temperatures and loading frequencies. These models use shear modulus |G*|, phase angle δ of the binder, and volumetric properties of asphalt mixture as input data. Artificial neural networks can also be used to predict asphalt properties tested in the DSR device (dynamic shear rheometer) [13].

The purpose of the present paper is to describe the development and use of a new model for predicting the stiffness modulus of high-modulus asphalt concrete (HMAC). The model is intended to calculate the stiffness modulus corresponding with a four-point bending beam (4PB-PR or 4PBB) determining method and it is based on the Witczak model [11]. It is important to notice that this model is intended for a four-point bending beam because the Witczak model is intended for another determining method called dynamic modulus (DM). To avoid misunderstanding, the laboratory methods of determining stiffness modulus were also described in papers [1,14]. An overview of the most important models for calculating stiffness modulus was also discussed in an earlier article [1].

The presented model is one of two models for stiffness modulus E_4PB_ calculation, which were developed as a result of a research project and study at Poznan University of Technology and the Laboratory of the General Directorate for National Roads and Motorways in Poznań, Poland.

## 2. Laboratory Research Program

In order to develop a predictive model to estimate the stiffness modulus of asphalt mixes, the following laboratory tests were planned to be performed:

1. Determination of stiffness modulus and phase angle of an asphalt mixture at various temperatures and various loading frequencies for each test temperature by the four-point bending beam test conducted according to [6];
2. Determination of shear modulus and phase angle of a binder at various temperatures and various loading frequencies for each test temperature by a dynamic shear rheometer test, carried out according to [15];
3. Determination of the resistance to hardening of binders under the influence of heat and air by an RTFOT test performed according to [16];
4. Determination of soluble binder content in an asphalt mixture conducted according to [17];
5. Determination of particle size distribution carried out according to [18];
6. Determination of the maximum density of an asphalt mixture according to [19];
7. Determination of bulk density of an asphalt mixture specimen according to [20];
8. Determination of air-voids content of an asphalt-mixture specimen according to [21];
9. Determination of dimensions of an asphalt-mixture specimen according to [22].

During the tests, every effort was made to meet the requirements of the above-mentioned standards.

## 3. Tested Asphalt Mixtures and Binders

**Figure 1:**
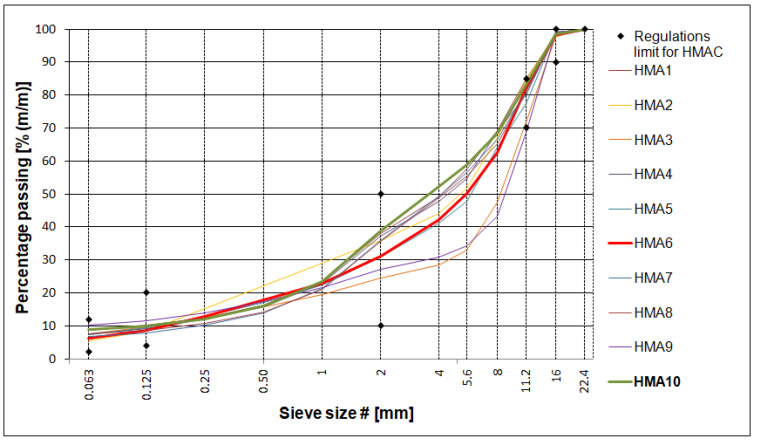
Gradation curves of aggregate mixtures.

Ten asphalt mixtures were tested as part of the research project. Three HMA (hot-mix asphalt) types were developed and tested specifically for the project, the other seven ones were tested as part of the standard activities of the Road Laboratory of the General Directorate of National Roads and Motorways in Poland. The testing program of the standard-tested HMA for the research project was extended. Most of the relevant data for the tested HMA are presented in Table 1. HMA mixtures were made with both unmodified and polymer-modified binders. Many research works concerning polymer-modified binders conducted previously by the authors were described in [23,24,25]. The gradation curves of aggregate mixtures used in each HMA are shown in Figure 1. The mixture with the lowest voids content is plotted in green (HMA 10) while the mixture with the highest voids content is plotted in red (HMA 6). The limits for the gradation curves are given in accordance with the Polish technical requirements for high-modulus asphalt concrete [26].

Among the ten HMA types tested, taking into account the Polish requirements [26], seven mixtures can be considered HMAC; these are mixtures in which mainly unmodified hard bitumen 20/30, or in one case with polymer modified bitumen PMB 25/55-60, were used. HMA 8 and HMA 10 mixtures contain a bituminous binder suitable for HMAC and the gradation curve also met the requirements specified for HMAC [26] but the values of stiffness modulus determined at 10 °C and 10 Hz were found to be less than E_4PB_ = 11,000 MPa, the minimum stiffness modulus value specified for HMAC for the base course, and, therefore, HMA 8 and HMA 10 mixtures, were recognized as asphalt concrete (AC). HMA 9, which is an SMA Jena mix (stone mastic asphalt used as a single-layer pavement), was decided to be included in the research program as a comparative mix. The six HMA mixtures (HMA 1–HMA 6) contain different 20/30 penetration-grade bitumen from three different manufacturers. HMA 7 and HMA 9 mixtures contain polymer-modified bitumen and HMA 8 and HMA 10 mixtures used PMB 25/55-80 highly modified bitumen (so-called HIMA), both from the same manufacturer. In all the tested HMA mixtures, limestone filler was used. An adhesion agent was added to all the tested HMA in an amount ranging from 0.3% to 0.5% by weight of the bitumen. Apart from the adhesive agent, no other modifiers were added to the HMA [27].

Finally, the results obtained for eight HMA mixtures (HMA 1-HMA 8) were used to develop the predictive model, of which seven were classified as HMAC and one (HMA 8) as asphalt concrete. Mixtures HMA 9 and HMA 10 were not included in the development of the predictive model, as their properties differed too much from the requirements specified for HMAC.

**Figure 2:**
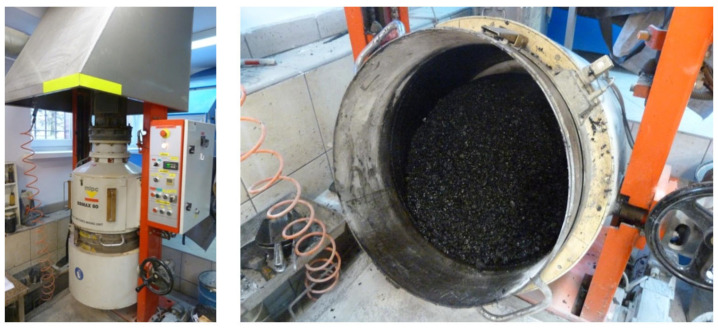
Laboratory mixer manufactured by MLPC.

**Figure 3:**
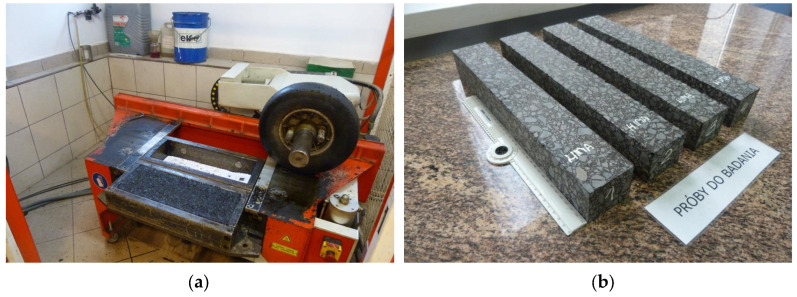
Photos taken during specimen preparation procedure: (**a**) Rolling device for compacting samples; (**b**) Samples prepared for testing.

The asphalt mixtures used in the study were prepared in a laboratory mixer (Figure 2), with the exception of HMA 6, which was taken from the site and transported to the laboratory. The mixing and paving temperatures were adopted depending on the type of mix and the type of bitumen used according to [26]. Two slabs of HMA from each mix were compacted in a roller device (Figure 3a) according to [28]. Four prismatic specimens (Figure 3b) with dimensions 50 mm × 60 mm × 380 mm (H × B × L) were cut from the compacted slabs, on which the stiffness modulus and phase angle could be determined using the four-point bending test (4PB-PR).

Each bitumen applied in the tested HMA was subjected to short-term RTFOT ageing in accordance with [16] prior to testing in the DSR.

## 4. Shear Modulus and Phase Angle of Bituminous Binders

**Figure 4:**
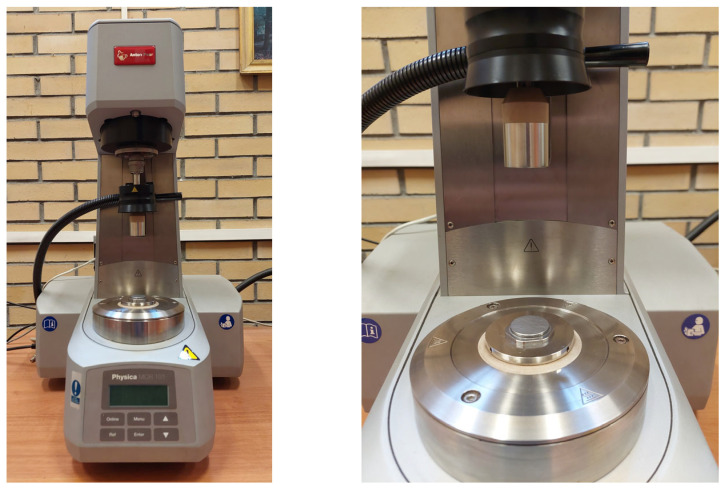
Dynamic Shear Rheometer [1].

The shear modulus and phase angle of the investigated bituminous binders were determined using a dynamic shear rheometer DSR, shown in Figure 4. Each type of bitumen used for the analyzed HMA was tested in the DSR. Tests were carried out at different temperatures and loading frequencies for each temperature so that the temperature and loading frequency on the DSR device were consistent with the temperature and loading frequency used in the determination of the stiffness modulus and phase angle of the HMA mixtures. The selection of test temperatures also took into account the limiting operating (equivalent) temperatures according to the Superpave classification. The temperatures and loading frequencies of the individual binders are shown in Table 2. In the first series, the tests were also carried out at high temperatures using a 25 mm diameter spindle. However, after analyzing the first series of tests, it became apparent that some of the test results may not meet the measurement uncertainty requirements specified in [15]. Therefore, it was decided to amend the test procedure and carry out a second series of tests. The second series of tests was carried out using only the 8 mm diameter spindle in the temperature range of −20 °C to 40 °C.

**Figure 5:**
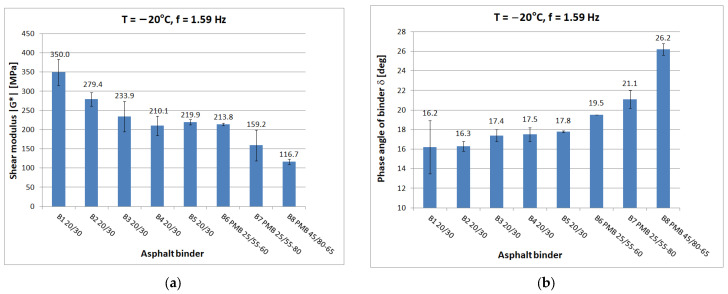
Dynamic Shear Rheometer bitumen test at T = −20 °C and f = 1.59 Hz: (**a**) Shear modulus |G*|; (**b**) Phase angle δ.

**Figure 6:**
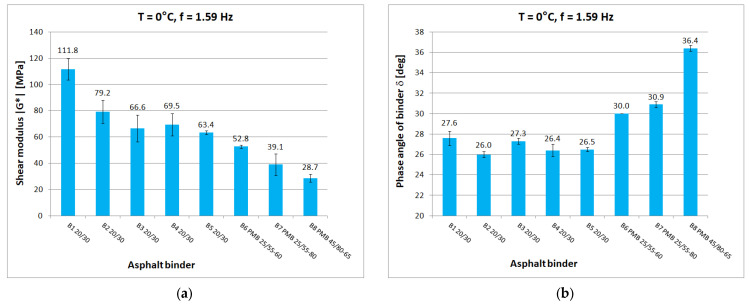
Dynamic Shear Rheometer bitumen test at T = 0 °C and f = 1.59 Hz: (**a**) Shear modulus |G*|; (**b**) Phase angle δ.

**Figure 7:**
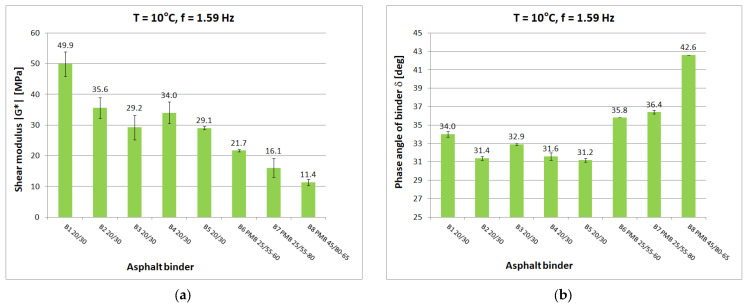
Dynamic Shear Rheometer bitumen test results at T = 10 °C and f = 1.59 Hz: (**a**) Shear modulus |G*|; (**b**) Phase angle δ.

**Figure 8:**
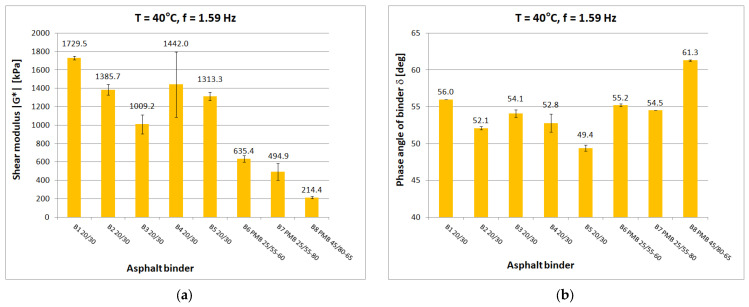
Dynamic Shear Rheometer bitumen test results at T = 40 °C and f = 1.59 Hz: (**a**) Shear modulus |G*|; (**b**) Phase angle δ.

**Figure 9:**
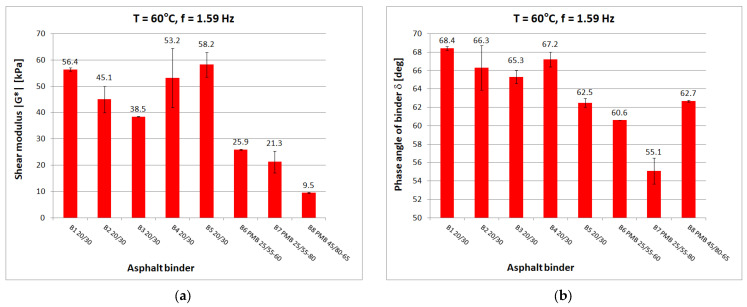
Dynamic Shear Rheometer bitumen test results at T = 60 °C and f = 1.59 Hz: (**a**) Shear modulus |G*|; (**b**) Phase angle δ.

Tests at each temperature and loading frequency were carried out on four bitumen samples. After a detailed review of the standards [15,29,30] for testing in the DSR apparatus, it was decided that the determinations of shear modulus and phase angle would be carried out from the highest to the lowest temperature. It was considered that the extreme test conditions, i.e., highest and lowest temperature and lowest and highest frequency, would be the most dangerous for the specimen which, after testing, was reflected in a higher measurement uncertainty for such conditions. When testing at high temperature, it was observed whether the specimen (at constant strain amplitude and maximum frequency) was still within the range of linear strain. Liquefaction and spillage of the sample on the lower measuring plate of the instrument resulted in the rejection of the test results. When conducting low-temperature tests, it was observed whether the sample still had proper adhesion to the lower measuring plate after the test. If it was found that adhesion had been lost, the results were discarded. Tests were carried out at a constant maximum swing angle value of θ = 0.001 rad in the frequency range of 0.1 Hz to 50 Hz. Tests were conducted from lowest to highest frequency. The temperature gradient was not greater than 1 °C/min. For each type of binder, the tests were carried out on four samples with a diameter of 8 mm and on four samples with a diameter of 25 mm. As a result of the test, the measurement system was saved to a .csv file on the control computer. Figure 5, Figure 6, Figure 7, Figure 8 and Figure 9 show chosen test results concerning shear modulus |G*| and phase angle δ of asphalt binders at loading frequency 1.59 Hz (10 rad/s).

During the preparation for the implementation of the study, the authors obtained information about the low reproducibility of test results obtained from DSR. Such news was also confirmed by the staff of the main laboratory of one of the companies. The data on the precision of the MSCR test available in the standard [30], i.e., a reproducibility of up to 43%, also did not inspire optimism. After carrying out the first series of tests, it turned out that the obtained results of the shear modulus |G*| were characterized by too high of a measurement uncertainty, significantly exceeding the 10% specified in [15] and did not meet the requirement for the arithmetic mean at the overlap temperature. It was decided to introduce changes to the DSR test procedure for the binders. Particular attention was paid to maintaining the correct specimen geometry and ensuring good adhesion of the binder to the rheometer test system by heating both test-system plates. These problems are mentioned in the European standard [15] but no specific solutions to these problems are given. After modifying the measurement procedure, a second batch of binder tests was carried out, with significantly less measurement uncertainty. The results obtained were assessed according to the reproducibility criteria contained in [15]. The criteria were considered to be fulfilled if the mean relative uncertainty RMU of the results of the determination of the shear modulus |G*| and the phase angle δ was less than 10% (for the shear modulus |G*|) and 5% (for the phase angle δ), respectively. The largest values of relative measurement uncertainty are obtained for extreme temperature and frequency conditions.

It is advisable to develop measurement-precision data for the entire spectrum of temperatures and frequencies (0.1 to 25 Hz), with a particular focus on frequencies of 1.59 Hz and 10 Hz.

The results were compared for the five selected temperatures used in the tests: −20 °C, 0 °C, 10 °C, 40 °C, and 60 °C. At low and medium temperatures, the results of the determined shear modulus can be divided in terms of obtained values into three groups:

- first group: B1 20/30 penetration grade bitumen with significantly higher values of shear modulus than other 20/30 bitumens;
- second group: binders B2 to B5, these are 20/30 penetration grade bitumens;
- third group: polymer-modified bitumen with the lowest values of shear modulus in this temperature range.

At 0 °C, the difference between the shear modulus determined for the B1 binder (111.8 MPa) and the average determined for the rest of the hard binders (69.7 MPa) is 42 MPa or 60% of the lower value. At high temperatures, the difference between the B1 binder and the other 20/30 unmodified binders disappears. However, it is still possible to observe clear differences in the values obtained for neat and polymer-modified binders. The shear modulus values decrease with increasing temperature and increase with increasing frequency, as can be observed in other studies. The obtained values confirm the fact that modified binders have a lower temperature susceptibility.

In the case of the phase angle of the tested binders, also at low and medium temperatures, a clear difference can be seen between the 20/30 unmodified binders with smaller phase angle values and the modified binders. At high temperature, on the other hand, it is the 20/30 unmodified binders that are characterized by larger phase-angle values. At 60 °C, the average value of the phase angle for hard binders (65.9°) is 8° higher than the average for polymer-modified binders (57.9°). The values of the phase angle of binders increase with increasing temperature and decrease with increasing frequency. The smallest value of the phase angle (16.2°) was obtained for B1 bitumen at −20 °C. The largest value of phase angle (68.4°) was also obtained for B1 bitumen at 60 °C.

## 5. Stiffness Modulus and Phase Angle of Asphalt Mixtures

**Figure 10:**
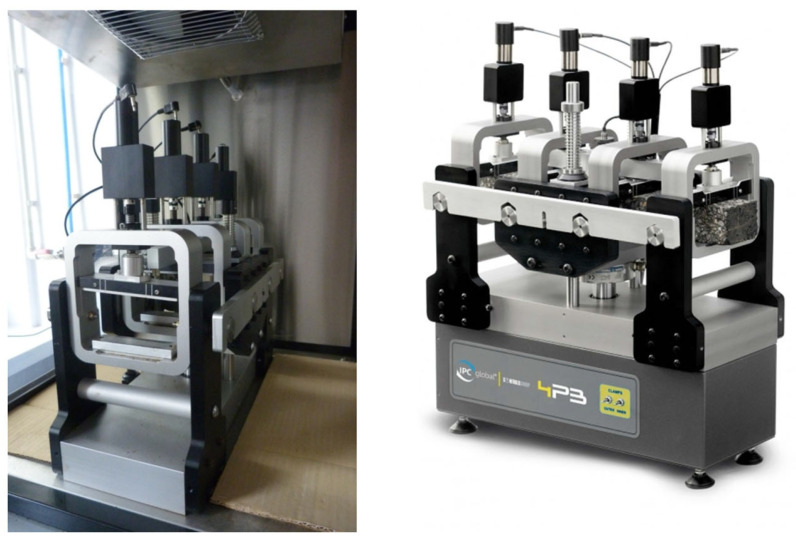
Four-point bending beam test device.

Determination of the stiffness modulus and phase angle of the HMA mixtures was carried out by using the 4PBB bending beam apparatus (Figure 10). The tests were performed at different temperatures and frequencies for each temperature. The variation in test temperature is intended to represent the different operating temperatures of the pavement. The different test frequencies also mimic the frequencies found in the pavement, which are primarily dependent on traffic speed.

The tests were carried out on 40 HMA beam samples made from 10 HMA mixtures (four samples per one HMA). All beam samples had the same dimensions, i.e., 50 mm × 60 mm × 380 mm (H × B × L). The specimens were conditioned from 4 to 6 h before the start of the test at each temperature. The execution of tests at different frequencies could be set in the test apparatus control program. The tests were carried out in controlled-strain mode, which in all cases was ε = 50 μm/m, thus avoiding fatigue processes. For each determination, 150 loading cycles were performed. Fifteen cycles (from 93 to 107 cycles) were used to calculate the values of the stiffness modulus E_4PB_ and the phase angle Φ_4PB_ of the asphalt mixture.

Temperature and frequency were not the same for each mixture due to technical reasons. Table 3 summarizes the temperature and loading frequency used in the tests for each HMA. The lowest temperature at which the mixtures were tested was 0 °C, as the climatic chamber of the testing machine did not allow a lower temperature to be set reliably. The highest temperature at which the mixtures were tested was 40 °C. According to [6], this is the highest temperature at which specimens should be tested due to the possibility of nonlinear deformation and the creep phenomenon of HMA. The tests were carried out from the lowest to the highest temperature and from the lowest to the highest frequency. For HMA 1, only the standard tests required by [26] were made. Due to the higher probability of nonlinear deformations, HMA 9 samples were not tested at 40 °C.

**Figure 11:**
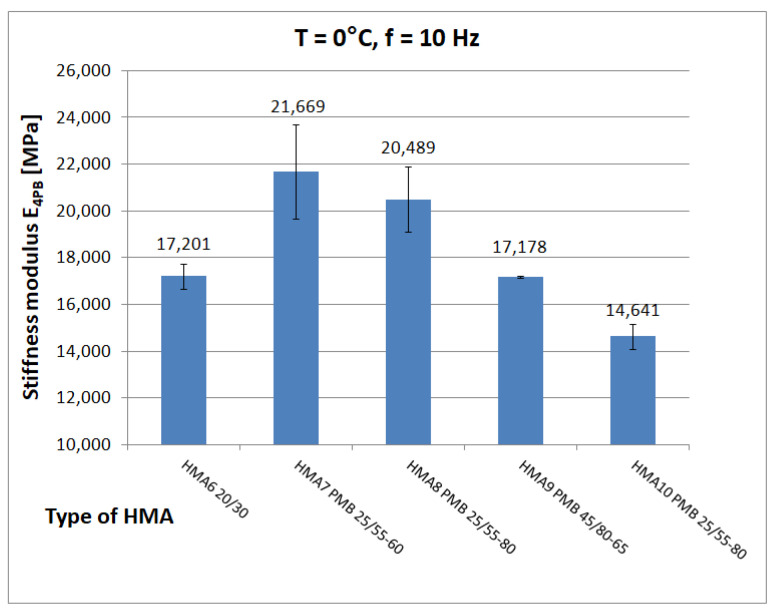
HMA Stiffness modulus test results at T = 0 °C and f = 10 Hz.

**Figure 12:**
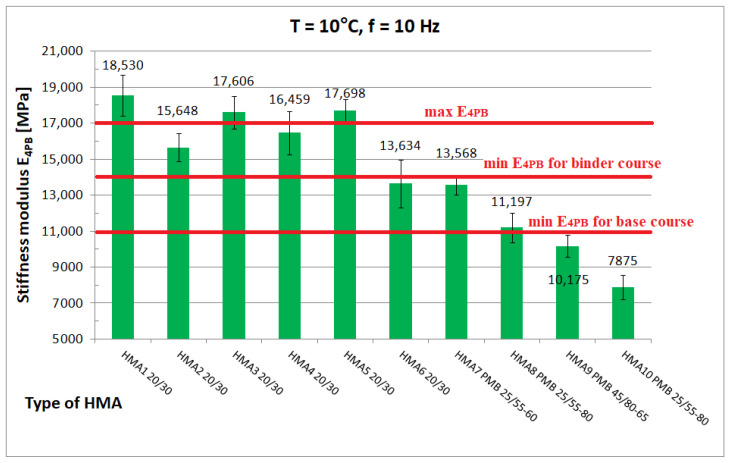
HMA Stiffness modulus test results at T = 10 °C and f = 10 Hz.

**Figure 13:**
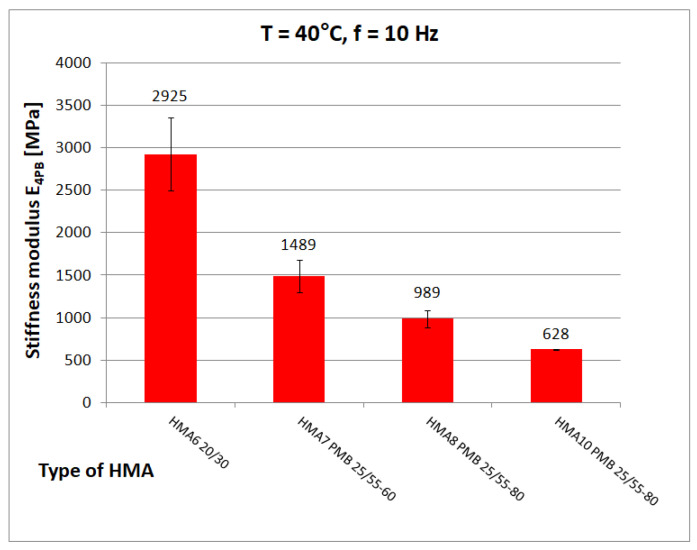
HMA Stiffness modulus test results at T = 40 °C and f = 10 Hz.

The tests discussed here yielded 855 determinations of the complex stiffness modulus E*, after excluding repetitive frequencies and files containing errors and the results obtained for HMA 9 and HMA 10 to develop an analytical-empirical model, 471 values of the complex stiffness modulus E* were obtained from the eight tested HMA. Figure 11, Figure 12 and Figure 13 show chosen test results of stiffness modulus |E*| of hot-mix asphalt for frequency 10 Hz at different temperatures. Figure 12 shows the limiting values of stiffness modulus |E*| specified in the Polish technical requirements [26].

The measurement results (expressed by mean value with uncertainty interval) of the stiffness modulus and phase angle were determined on a sample of 15 values. The largest measurement uncertainty values (approximately 10 MPa) were found in the case of the tests carried out at the lowest applied frequency of 0.1 Hz. All mean relative measurement uncertainty values were less than 1%.

When the precision of the determination of the stiffness modulus and the phase angle using the four-point bending beam method (4PBB) is considered, it should be borne in mind that a significant source of uncertainty is the way the specimen is mounted in the test device. This was also mentioned in [31]. The value of the measurement uncertainty of these quantities is also influenced by the fact that the algorithm for approximation of the measured data is not specified in [6], and the possible different possibilities of calculating the result of the determination (using the approximation, or directly from the measured values) that result from this fact.

At low temperature (0 °C), the higher value of the stiffness modulus E_4PB_ = 21,080 MPa (mean) is achieved by HMA 7 and HMA 8 with a modified binder with 5.2% bitumen content by weight, while HMA 6 with an unmodified binder with the same bitumen content by weight has a significantly lower (by 3880 MPa) value of the stiffness modulus E_4PB_ = 17,200 ± 548 MPa, which is, according to the authors’ opinion, an unusual behaviour. At medium temperature (10 °C), the highest value of stiffness modulus E_4PB_ = 18,530 ± 1137 MPa is achieved by HMA 1, which corresponds well with the value of shear modulus of B1 bitumen. According to statistical tests performed at 10 °C, the stiffness modulus E_4PB_ of the HMA 1 mix has a higher value than that of HMA 3 (17,606 ± 907 MPa), HMA 4 (16,459 ± 1219 MPa), and HMA 5 (17,698 ± 613 MPa) mixes. In contrast, the E_4PB_ stiffness modulus values of the HMA 3 and HMA 5 mixtures are equal to each other. However, the relative difference (5%) between the E_4PB_ stiffness modulus values of HMA 1 and HMA 3 and HMA 5 is much smaller than the relative difference (75%) between the shear modulus values of the bitumen used as a binder, as B1 bitumen has a significantly higher shear modulus value |G*|. This can be explained by the higher volumetric content of bitumen in HMA 1 and the differences in voids content V_a_. At 40 °C, HMA 6 with 20/30, bitumen stands out with a significantly higher value of stiffness modulus E_4PB_ = 2925 ± 429 MPa than mixtures with polymer-modified bitumens (average E_4PB_ = 1239 MPa).

## 6. Effective Bitumen Content, Air Voids, and Granulation of HMA

**Figure 14:**
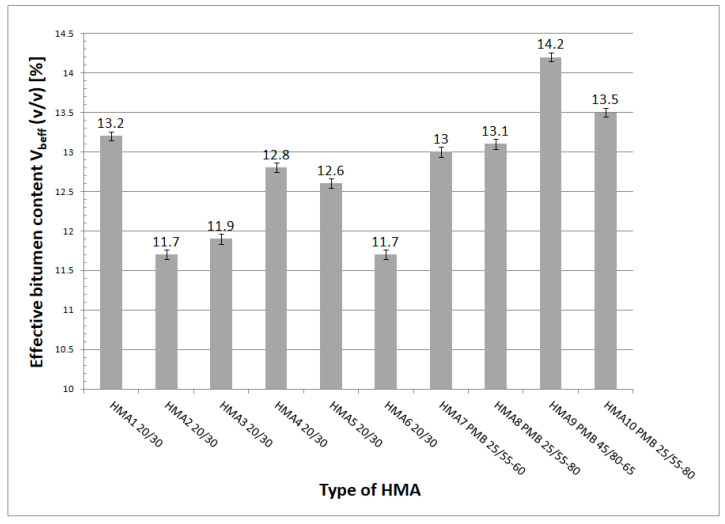
Effective bitumen content V_beff_ values.

One of the variables used in the equation of the Witczak–El-Badawy model is the effective bitumen content in the HMA V_beff_. In order to calculate the value of this variable, it is necessary to determine the density of bitumen G_b_ used in the HMA. The authors adopted the values of bitumen density G_b_ from data made available by bitumen manufacturers. The results of the effective bitumen content V_beff_ obtained from tests carried out on the basis of [17] are shown in Figure 14.

**Figure 15:**
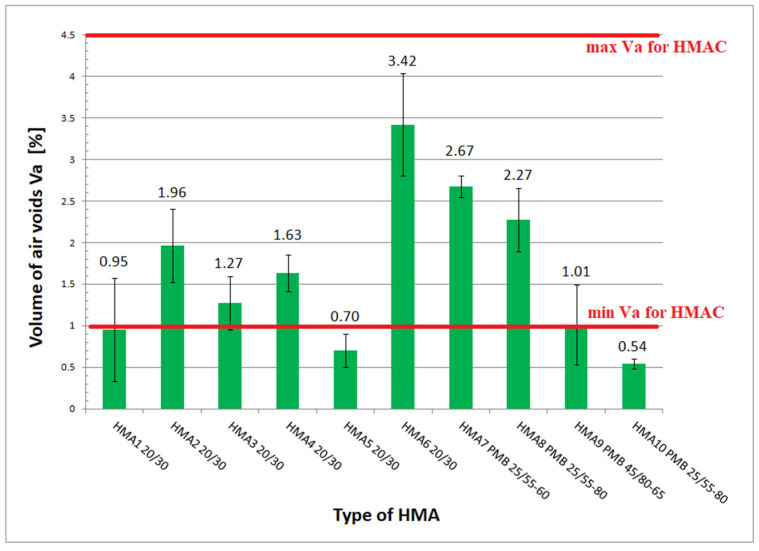
Air-voids content V_a_ in HMA.

From the sensitivity analysis performed according to [11], it appears that the value of the stiffness modulus depends on the air-voids content of the HMA. In view of the mixes with the lowest homogeneity, it was decided that the stiffness modulus values marked on the individual beams with their corresponding air-voids contents would be used to optimize the predictive-model coefficients. The bulk density determined by method B (in water) was used to calculate the air-voids content. The bulk density of the specimens can also be determined using method D (from sample dimensions). However, on the basis of the tests carried out, it can be assumed that due to the small difference in values, the determination method has no influence on the bulk density value. The results of the air-voids content obtained from the tests [20] using method B (in water) are presented in Figure 15. This figure shows also the limiting values of air voids Va specified in the Polish technical requirements [23].

The granulation data was calculated based on the gradation-curve values determined for the HMA used for the beam specimens (Table 4). Explanations of the grain-size data are given next to Equation (2).

## 7. Predictive Model

Nowadays the most commonly used models are the Witczak model [11,32], the Hirsch model [33,34] and models using artificial neural networks (ANN models) [35,36,37]. New predictive models are also constantly being developed [38,39,40]. A new predictive model for calculating the stiffness modulus for the four-point bending beam method (4PBB) was developed based on a form of the Witczak–El-Badawy model equation for the dynamic-modulus method (DM), commonly used in countries designing pavements according to MEPDG. The results of the Witczak–El-Badawy model are presented in [41]. The equation of this model is presented as Equation (1).

(1) $$\begin{array}{cc} {log\;E}_{DM}^{}=0.02 & +\;0.758\cdot \left({\left|G^{*}\right|}^{-0.0009}\right)\\ & \cdot \;[6.8232-0.03274\cdot P_{200}+0.00431\cdot {P_{200}}^{2}+0.0104\cdot P_{4}-0.00012\cdot {P_{4}}^{2}+0.00678\cdot P_{38}\\ & -\;0.00016\cdot {P_{38}}^{2}-0.0796\cdot V_{a}-1.1689\cdot \frac{V_{beff}}{V_{beff}+V_{a}}]\\ & +\;\frac{1.437+0.03313\cdot V_{a}+0.6926\cdot \frac{V_{beff}}{V_{beff}+V_{a}}+0.00891\cdot P_{38}-0.00007\cdot {P_{38}}^{2}-0.0081\cdot P_{34}}{1+exp\left[-4.5868-0.8176log\left|G^{*}\right|+3.2738log\delta \right]} \end{array}$$

where:

E_DM_—stiffness modulus determined by dynamic-modulus test [10^5^ psi];

|G*|—shear modulus of binder [psi];

δ—phase angle of binder [°];

V_beff_—effective binder content expressed by volume (*v*/*v*) [%];

V_a_—air-voids content [%];

P_200_—percentage of aggregate passing the No. 200 sieve (#0.075 mm) [%];

P_4_—cumulative percentage retained on the No. 4 sieve (#4.76 mm) [%];

P_38_—cumulative percentage retained on the 3/8 in sieve (#9.5 mm) [%];

P_34_—cumulative percentage retained on the 3/4 in a sieve (#19 mm) [%].

However, the form of the model equation (Equation (1)) has undergone some modifications, also aimed at simplification. Some quotients (four) with the squares of the variables characterising the aggregate grain-size distribution were removed from the Witczak–El-Badawy model equation but other quotients with the variables characterizing the aggregate-size grain distribution were still left. The decision to remove some quotients from the equation was based on the results of the sensitivity analysis carried out for the Witczak–Bari model presented in [11]. This analysis shows that the variables with the smallest impact on the equation value are precisely the variables characterizing the aggregate grain size. In addition, the grain-size and air-voids content of HMA are the dependent variables and changes in grain size are partly taken into account by changing the air-voids content in HMA. Removing the quotients from the equation significantly simplifies both the model equation and the process of optimising the coefficients of the modified equation. Furthermore, the simplified model with 17 coefficients gave better fitting quality parameters than the Witczak–El-Badawy model equation with 21 coefficients. The equation of the modified model is presented as Equation (2). After these modifications, the model was named model A. The types of sieves used to characterize the grain size of HMA were also modified. In the original Witczak–El-Badawy model equation, sieves typical of AASHTO-associated countries, such as the sieve with a mesh side dimension of 0.075 mm, were used. This set of sieves has been replaced by a set used in some European countries (e.g., in Poland), with a sieve mesh side size as close as possible to the sieves being replaced.

(2) $$\begin{array}{c} {logE}_{4PB}^{}=A1+A2\cdot \left({\left|G^{*}\right|}^{A3}\right)\cdot \left[A4+A13\cdot P_{0}+A14\cdot P_{4}+A15\cdot P_{8}+A9\cdot V_{a}+A10\cdot \frac{V_{beff}}{V_{beff}+V_{a}}\right]\\ +\frac{A5+A11\cdot V_{a}+A12\cdot \frac{V_{beff}}{V_{beff}+V_{a}}+A16\cdot P_{8}+A17\cdot P_{16}}{1+exp\left[A6+A7log\left|G^{*}\right|+A8log\delta \right]} \end{array}$$

where:

E_4PB_—stiffness modulus determined by four-point bending beam test [MPa];

|G*|—shear modulus of binder [MPa];

δ—phase angle of binder [°];

V_beff_—effective binder content expressed by volume (*v/v*) [%];

V_a_—air-voids content [%];

P_0_—percentage of aggregate passing the 0.063 mm sieve [%];

P_4_—cumulative percentage retained on the 4.0 mm sieve [%];

P_8_—cumulative percentage retained on the 8.0 mm sieve [%];

P_16_—cumulative percentage retained on the 16.0 mm sieve [%];

A1–A17—optimized equation coefficients [-].

As already stated, 471 values of the stiffness modulus E_4PB_ and the phase angle Φ representing the results of determinations made on the eight HMA mixtures were used to develop model A. The range of variability of the input data for the optimization process, i.e., the variables in the models, is given in Table 5.

Optimization of the coefficients in the equations of the models was carried out using the lsqnonlin command in Matlab, which returns the solution of the nonlinear least-squares method task. The least-squares method is a method of approximating a function of a given type, to a set of empirical points. The method consists in choosing the parameters of the function being approximated in such a way that the sum of squares of the deviations of the empirical points from the value of this function is as small as possible.

Optimization was carried out both by specifying boundary conditions and without specifying them. In the case of model A, the solution depended on boundary conditions, similar to the authors of this model [32]. The optimization process for model A was carried out from a very large number of starting points determined in different ways, also by drawing the values of boundary conditions from a given interval. The evaluation of the solution took into account the value of the sum of least squares (∑e_i_^2^), the limiting value of relative errors (P95(re)) and the distribution of absolute errors (Table 6). As one can read in the work [32], it was discussed that in order to be considered a valid model, the distribution of absolute errors, after discarding outliers (nout), should be a normal distribution, and it was considered that the normality of the distribution should be confirmed by both histogram and statistical tests. The coefficients calculated as a result of the optimization procedure were presented in Equation (3) (after inserting them into Equation (2) of model A).


(3) $$\begin{array}{cc} {logE}_{4PB}^{}=-5.514 & +\;0.694\cdot \left({\left|G^{*}\right|}^{0.0273}\right)\\ & \cdot \left[10.2-0.0422\cdot P_{0}-0.0002\cdot P_{4}-0.0146\cdot P_{8}-0.16\cdot V_{a}-1.87\cdot \frac{V_{beff}}{V_{beff}+V_{a}}\right]\\ & +\frac{2.33+0.07\cdot V_{a}+1.23\cdot \frac{V_{beff}}{V_{beff}+V_{a}}+0.014\cdot P_{8}-0.0009\cdot P_{16}}{1+exp\left[-7.734-0.6795log\left|G^{*}\right|+3.4156log\delta \right]} \end{array}$$

Parameters used in Equation (3) were explained under Equation (2).

**Figure 16:**
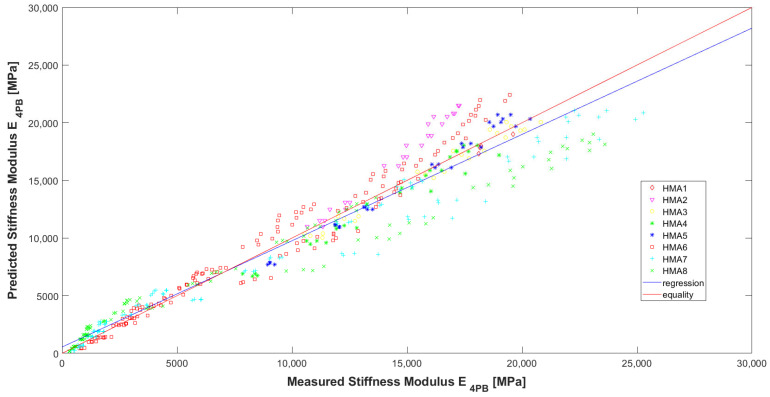
Comparison between measured and calculated values of the stiffness modulus E_4PB_ of HMA with marked types of asphalt mixtures.

**Figure 17:**
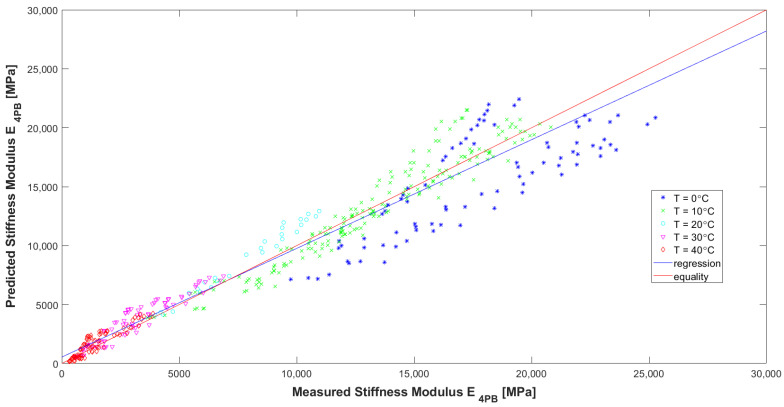
Comparison between measured and calculated values of the stiffness modulus E_4PB_ of HMA with marked temperatures used in the tests.

**Figure 18:**
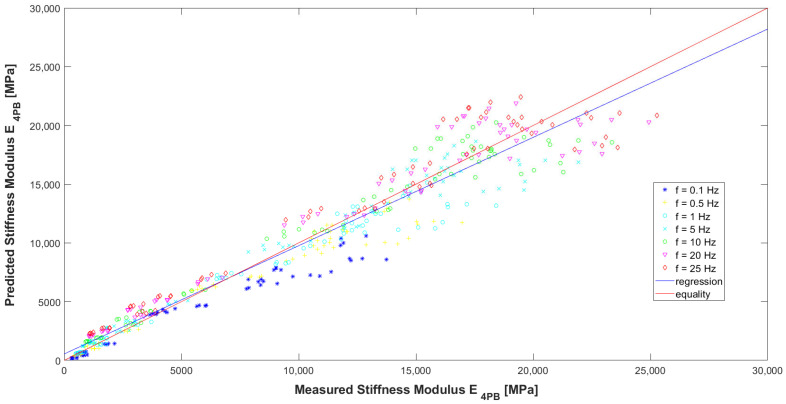
Comparison between measured and calculated values of the stiffness modulus E_4PB_ of HMA with marked loading frequencies used in the tests.

Evaluating the model according to the graphs (Figure 16, Figure 17 and Figure 18) and Table 7, it can be concluded that it has high precision and low bias (indicators explained in [32]). In Figure 16, it can be seen that for the HMA 7 and HMA 8 mixes, the determined E_4PB_ stiffness moduli are greater than the calculated ones, while the opposite is true for the HMA 2 and HMA 6 mixes. The influence of the type of aggregate and, more specifically, the silica content (SiO_2_) in the aggregate, which affects the adhesion of the asphalt to the aggregate, can be seen here. For mixtures with more alkaline aggregates (HMA 7 and HMA 8), the determined stiffness moduli of E_4PB_ are larger than calculated. Therefore, at the stage of further work on the predictive models, the introduction of an additional variable characterizing the silica content of the aggregate used to make the HMA can be considered in the models. In Figure 17, where the test temperatures T are marked, it can be clearly seen that for high temperature, lower values of the stiffness modulus E_4PB_ were obtained, and for low temperature, higher values of the stiffness modulus E_4PB_ were obtained, confirming the generally known relationship and confirming the correctness of the developed model A. It can be noted, however, that for the analysed HMA, the values of stiffness moduli E_4PB_ obtained at 10 °C are only slightly smaller than those obtained at 0 °C. In Figure 18, where the loading frequencies f are marked, no significant relationship is noticed.

To further confirm the validity of the developed model for calculating the E_4PB_ stiffness modulus, a normality analysis of the distribution of the model’s absolute errors was performed. The analysis of the normality of the distribution was performed by plotting histograms and performing multiple statistical tests for the normality of the distribution, with a significance level of α = 0.05. The D’Agostino–Pearson test was used as the binding test for the normality of the distribution due to the fact that the power of this test classifies at the medium level. Before analyzing normality, deviating absolute error values were rejected using the Hampel statistical test.

**Figure 19:**
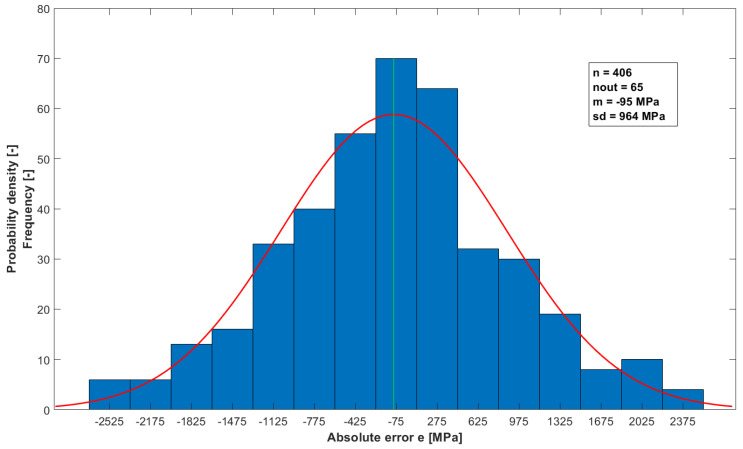
Histogram of absolute errors of model A.

After applying the Hampel statistical test to eliminate outliers from the set of absolute errors for model A, 65 outliers were eliminated. The histogram (Figure 19) indicates that the distribution of errors may be a normal distribution. The results of the applied statistical tests for the normality of the distribution are summarized in Table 8. The distribution of errors can be considered normal when the value of the *p* statistic > 0.05. According to all applied tests, the distribution of absolute errors can be considered a normal distribution. Almost all outliers, with few exceptions, are values calculated for results obtained at 0 °C.

**Figure 20:**
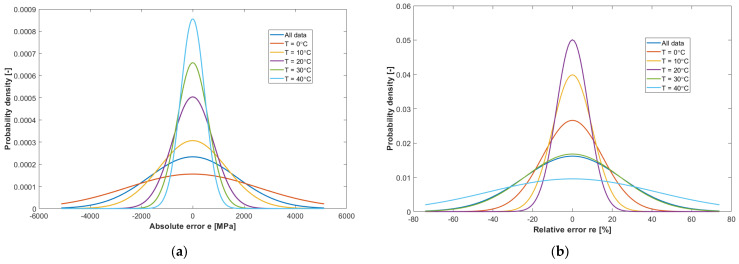
Error distributions of model A for different temperatures: (**a**) Distribution of absolute errors; (**b**) Distribution of relative errors.

Figure 20a,b show the distributions of the absolute and relative errors of model A, for different temperatures at which the E_4PB_ stiffness modulus determinations were carried out. The highest values of absolute errors are shown by determinations of the E_4PB_ stiffness modulus made at 0 °C, while the lowest values of absolute errors are shown by tests carried out at the highest temperature of 40 °C. Such error distributions are consistent with the achieved E_4PB_ stiffness modulus values. The largest relative error values are shown by the E_4PB_ stiffness modulus determined at 40 °C and 30 °C, where the values are so small that even a small difference will result in a significant relative error. Medium relative error values were found in the results of tests carried out at 0 °C. The smallest relative error values were found at 10 °C and 20 °C. The relative error values at 10 °C are within approx. 30% and at 20 °C within approx. 20%, which can be considered satisfactory.

## 8. Concluding Remarks

According to the study and its analysis, further search for a better formulation of the model equation may not yield significant results. However, the introduction of an additional variable into the models to determine the silica content of the aggregate used to make the HMA or variables characterizing the shape of the aggregate grains could be considered. Further efforts to increase the accuracy of predictive models should focus on reducing the measurement uncertainty of the variables used in the models, which would be in line with the work described in [34].

It is also very important to assess predictive models in terms of relative errors. It can be concluded that in the literature on these models, information containing relative error analysis is very scarce. Among many publications analyzed, the authors found information on relative errors only in the article [34], where the figure includes simple ones specifying a relative error of re = 50%. Model A developed in this paper has just such a value for relative errors (P95(re) = 50%). The absolute errors of model A have a normal distribution similar to the model described in the work [11]. The correlation between the values obtained from the laboratory tests (Measured E_4PB_) and the values obtained from the model (Predicted E_4PB_) is very high, the coefficient of determination being R^2^ = 0.936.

Model A shows the greatest inaccuracy in calculating the stiffness modulus E_4PB_ at 0 °C, confirming the results of the work of Bari and Witczak [32]. Similar to the mentioned researchers, the authors look for physical hardening effects here.

The authors are of the opinion that, in order to increase the accuracy of model A, a number of measures are needed to reduce the uncertainty of both the input variables of the model in question (especially a reduction in the uncertainty of the shear modulus |G*| of the bitumen) and the stiffness modulus E_4PB_ of HMA itself. A more precise procedure for determining the stiffness modulus E_4PB_ would contribute to a reduction in uncertainty. The conditions for thermostating the specimens prior to the low-temperature determination of the stiffness modulus E_4PB_ of HMA need to be defined more precisely in order to further reduce the possible influence of physical hardening. The algorithm for calculating the stiffness modulus E_4PB_ of HMA also needs to be clearly defined.

Using model A, it is proposed that, in addition to calculating the value of the HMA stiffness modulus E_4PB_, it is also proposed to calculate the limiting values at the significance level α = 0.05 within which the result of the determination should fall, i.e., the uncertainty interval. These limits are proposed to be calculated using the P95(re) statistic. This would be done by subtracting and adding 50% of this value from the calculated value of the stiffness modulus E_4PB_ of the HMA; in this case, it would be known in which interval with 95% probability the laboratory-determined value of the stiffness modulus E_4PB_ should fall.

## Figures and Tables

**Table 1:** Characteristics of the tested hot-mix asphalts.

HMA ID	Type of HMA	Type of Bitumen	Bitumen ID	Effective Content of Bitumen P_be_ (m/m) [%]	Type of Aggregate Used in HMA
HMA 1	HMAC 16	20/30	B1	5.3	basalt
HMA 2	HMAC 16	20/30	B2	5.2	melaphyre serpentine granite
HMA 3	HMAC 16	20/30	B3	5.2	greywacke granite
HMA 4	HMAC 16	20/30	B4	5.3	basalt limestone
HMA 5	HMAC 16	20/30	B4	5.2	granodiorite limestone basalt
HMA 6	HMAC 16	20/30	B5	5.2	granite
HMA 7	HMAC 16	PMB 25/55-60 (modified)	B6	5.2	basalt
HMA 8	AC 16	PMB 25/55-80 (highly modified)	B7	5.2	basalt
HMA 9	SMA Jena 16	PMB 45/80-65 (modified)	B8	5.6	granodiorite
HMA 10	AC 16	PMB 25/55-80 (highly modified)	B7	5.6	basalt

**Table 2:** Temperature and loading frequencies set in the DSR device.

**Temperature T [°C]**	−20, −10, 0, 10, 30, 40, 60, 70, 80
**Frequency f [Hz]**	0.1, 0.2, 0.5, 1, 1.59, 5, 10, 20, 25, 50

**Table 3:** Summary of temperature and frequency values used in HMA tests performed using the 4PBB device.

HMA ID	Temperature [°C]	Frequency [Hz]
HMA 1	10	10
HMA 2	10	0.5; 1; 5; 10; 20; 25
HMA 3	10	0.5; 1; 5; 10; 20; 25
HMA 4	10	0.1; 0.5; 1; 5; 10; 20; 25
HMA 5	10	0.1; 0.5; 1; 5; 10; 20; 25
HMA 6	0; 10; 20; 30; 40	0.1; 0.5; 1; 5; 10; 20; 25
HMA 7	0; 10; 30; 40	0.1; 0.5; 1; 5; 10; 20; 25
HMA 8	0; 10; 30; 40	0.1; 0.5; 1; 5; 10; 20; 25
HMA 9	0; 10; 30	0.1; 0.5; 1; 5; 10; 20; 25
HMA 10	0; 10; 30; 40	0.1; 0.5; 1; 5; 10; 20; 25

**Table 4:** The mineral-aggregates granulation data.

HMA ID	P_0_ [%]	P_4_ [%]	P_8_ [%]	P_16_ [%]
HMA 1	8.7	50.9	30.7	1.6
HMA 2	5.6	55.9	32.8	1.3
HMA 3	7.6	71.4	52.5	2.1
HMA 4	7.6	52.2	31.1	0.8
HMA 5	7.2	58.9	35.8	1.0
HMA 6	6.4	57.7	37.3	1.7
HMA 7	6.7	50.5	33.4	1.5
HMA 8	7.5	51.1	34.8	1.8
HMA 9	8.7	50.9	30.7	1.6
HMA 10	5.6	55.9	32.8	1.3

**Table 5:** Range of variability of the variables used to develop model A.

Variable		Minimum	Maximum	Average
|E*|	[MPa]	290	25,268	9489
Φ	[°]	3.85	48.92	19.08
|G*|	[MPa]	0.09	133.65	30.78
δ	[°]	22.00	59.50	38.59
V_beff_	[%]	11.6	13.5	12.6
V_a_	[%]	0.5	3.8	2.2
P_0_	[%]	5.6	10.2	7.4
P_4_	[%]	50.5	71.4	56.9
P_8_	[%]	30.7	56.6	39.0
P_16_	[%]	0.8	2.1	1.5

**Table 6:** Chosen results of optimisation of the coefficients of model A.

$\sum {e_{i}}^{2}$	P95(re)	Nout
2,755,451,769	66	41
1,984,652,225	52	59
3,337,871,386	64	56
4,276,307,701	58	82
1,394,365,412	50	65

**Table 7:** Comparison of the quality of fit of model A.

Parameter		Value for Model A
No. of data points, n		471
No. of mixes, n_m_		8
Σe	[MPa]	100,538
Σ|e|	[MPa]	556,788
m(|e|)	[MPa]	1182
m(re)	[%]	−3
P95(re)	[%]	50
Se	[MPa]	1722
SDy	[MPa]	6826
Se/SDy	[-]	0.25
R^2^	[-]	0.936
aR^2^	[-]	0.934

**Table 8:** Results of statistical tests for normality of distribution.

Type of Test	The *p*-Value Statistic
Kolmogorov–Smirnov test	0.5664
Lilliefors test	0.1488
Shapiro–Wilk test	0.1901
D’Agostino–Pearson test	0.9475

## Data Availability

Data sharing is not applicable to this article.
